# Association between family environment and attention deficit hyperactivity disorder in children – mothers’ and teachers’ views

**DOI:** 10.1186/1471-244X-13-215

**Published:** 2013-08-27

**Authors:** Thiago de Oliveira Pires, Cosme Marcelo Furtado Passos da Silva, Simone Gonçalves de Assis

**Affiliations:** 1 Latin-American Centre for Studies on Violence and Health (CLAVES), National School of Public Health, Oswaldo Cruz Foundation, Av. Brasil 4036 - sala 700, Manguinhos, 21040-361 Rio de Janeiro, RJ, Brazil; 2 Department of Epidemiology and Quantitative Methods in Health, National School of Public Health, Oswaldo Cruz Foundation, Rio de Janeiro, RJ, Brazil

**Keywords:** ADHD, Family environment, Perinatal period, Hierarchical model

## Abstract

**Background:**

To ascertain whether factors of the family environment and gestational period are associated with the appearance of ADHD in children, as reported by various different informants (mothers and teachers).

**Methods:**

This paper presents results from the dataset of a longitudinal study to evaluate behavioral problems among schoolchildren in São Gonçalo, Rio de Janeiro State, in 2005 and 2006. The cross-section considered for this paper comprises records of exposure factors and ADHD. In all, 370 schoolchildren of the public school system were assessed by 3-stage cluster sampling. The Child Behavior Checklist (CBCL) and the Teacher Report Form (TRF) were used to measure outcomes. The exposure factors examined were: profile of child and mother, variables relating to the family environment, and perinatal considerations. The questions were answered by mothers and teachers. A hierarchical logistic regression model was used.

**Results:**

Precariously functioning families, lack of social support for mothers, adverse life events and discord during pregnancy were the factors associated with mother-reported ADHD. When ADHD was reported by teachers, the variables selected were: Intelligence quotient (IQ) and sex, with children with low IQ scores and boys more likely to display the disorder.

**Conclusions:**

Assessment of ADHD by teachers or mothers reveals specific characteristics that reflect how each of these informants understands the children. This highlights the importance of using informants from different environments in diagnosing the disorder.

## Background

The first systematic explanation of Attention Deficit Hyperactivity Disorder (ADHD) appeared in 1902 in a description of the history of 20 children whose symptoms were similar to those that today we call hyperactivity [1]. At present, the problem is defined as ADHD (Diagnostic and Statistical Manual of Mental Disorders - DSM-IV-TR™ 2000) and characterized as a consistent pattern of lack of attention and/or hyperactivity that is more frequent and severe than typically observed in individuals at an equivalent level of development.

Although the origin of the disorder is explained substantially by genetics [2,3], interaction between hereditary factors and environmental factors or psychosocial agents [1] is considered relevant to the causality of ADHD. On that view, genetics is considered to influence the likelihood that parents/guardians will construct environments favorable to the disorder’s manifesting in children [4]. In that regard, the importance of factors connected with the family environment in the etiology of ADHD has been described [5-7]. One example are Rutter’s adversity events [8], which point to a combination of a set of family environment factors (including severe interparental discord, low-income class, numerous families, paternal criminality, maternal mental disorder, and children raised under adoption conditions) being strongly related to psychological disorders, including ADHD [9]. In addition to these factors, deaths in the family, parents’ loss of employment [5], precarious social support, deficient family functioning, and the presence of substantial stressors in the family [6] were also associated with ADHD.

Another aspect important to understanding ADHD relates to behavior and problems experienced by the mother during pregnancy. On this line of etiology, some studies indicate that tobacco and alcohol use and psychological stress during pregnancy increase the risk of the child’s developing ADHD [10,11].

ADHD is one of the commonest childhood disorders, occurring in 3% to 7% of school-age children on clinical diagnosis. Although clinical assessment is considered the yardstick for diagnosis of psychiatric disorders, clinical psychiatrists are little used in epidemiological studies for practical and financial reasons [1]. In population studies using screening measures, estimates range from 2.3% to 19.8% [12]. Research with students 4–18 years in Turkey using the Child Behavior Checklist (CBCL) and Teacher Report Form (TRF) found the prevalence of 1.6% and 2.4% respectively [13]. Diverse factors influence the various estimates of prevalence, such as case definition criterion not always following DSM-IV criteria, which include the duration of the symptoms and clinically significant harm to the child’s life [14-16]. Another criterion mentioned relates to how persistently the problem is detected in different contexts. Accordingly, information from parents and teachers, in combination or independently, has been used in ADHD screening studies, thus covering the two main contexts of children’s lives [17,18]. Studies that rely on only one informant type tend to overestimate prevalence [12].

However, it is usual for there to be some degree of discrepancy between parents’ and teachers’ assessments of the behavior of children with ADHD [19]. In this respect, teachers tend to identify the disorder more often than parents. Factors such as classroom organization, which makes greater demands on the child’s ability to pay attention, as well as greater control being exerted over the children in the classroom, may explain these discrepancies [1]. In addition, teachers have a better grasp of appropriate behavioral development [18]; their assessments are more strongly associated with neurocognitive correlates [20]; and they perceive ADHD symptoms better in boys than in girls [21]. All the same, aspects such as class size and pupils’ cultural background have an influence on teachers’ diagnosis. Albuquerque and Oliveira [22] claim that parents’ and guardians’ reporting of ADHD symptoms is more inconsistent than teachers’.

Few studies have linked environmental factors to ADHD through the perspective of different informants. An example is the article of Kollins et al. [23], which analyzes the exposure of postnatal smoking by parents with ADHD according to the vision of parents and teachers. The researchers showed that postnatal parental smoking was associated with both parent and teacher ratings of ADHD symptoms.

This paper aims to ascertain whether factors relating to family environment and gestational period are associated with the appearance of ADHD in children and if these factors vary according to different informants (mothers and teachers). Despite the importance of the subject, there has been little discussion in Brazil about how environmental and psychosocial factors relate to ADHD [7]. Prior to this paper, no studies that evaluate how assessment by mothers and teachers interferes in this relationship were identified in Brazil.

## Methods

This paper presents baseline results from a 2005 longitudinal study measuring exposure factors and ADHD.

### Sample

The sampling plan was based on a record of public schools, classes and mean number of pupils per class provided by the São Gonçalo municipal education department for 2005 (universe of 6,589 first-year pupils of fundamental schooling). The sampling design employed is the three-stage cluster selection type (schools, first-year classes, and pupils). Random selection of the 25 schools was by systematic probability proportional to size (PPS) sampling; two classes were drawn at random from each school, and 10 pupils from each class, totaling 500 pupils in the sample. Two chances were given for the mother or guardian to attend the interview at the school. In the case of non-attendance after two opportunities, or if the pupil had left the school, a new interviewee was summoned according to the prior draw. 1% of participants recruited refused to take part in the study, and approximately 35% of the pupils originally selected were replaced by the next candidate on the list for their class, mainly due to faulty class record keeping.

In this paper, 370 children are assessed (4935 in the expanded sample). The 130 exclusions responded to the following criteria: children with intelligence quotient (IQ) ≤ 69, given the difficulty of assessing ADHD in children with such a low intellect score [24]; one child the test could not be applied to; and 109 children whose informant was not their mother or whose ADHD was not assessed by their teacher.

The group of losses and the group selected were compared by analysis by sex, age and social class, but no statistically significant differences were encountered (p < 0.05).

### Instruments

ADHD’symptoms was assessed using the CBCL and the TRF. Both instruments were formulated by the same authors [25], and diagnosis was based on their own criteria.

Brasil and Bordin [26] shows that the CBCL (externalizing/internalizing/total problems scales) has significant correlation with the PL-KSADS (clinical) in a brazilian sample characterized by low maternal education and families with low standard of living.

The CBCL evaluates behavioral problems occurring in the prior six months in children 6–18 years old, on the basis of information given by their parents. Specifically as regards the inattentive and hyperactive behavior considered in this paper, informants responded to 10 items, including questions such as whether the child is restless, never still; is impulsive, acts without thinking; can’t manage to finish what it starts; and is easily distracted, unable to pay attention for long. The answer options varied from false to very true (0 to 2 points). The items were added and standardized so that they had a mean of 50 and standard-deviation of 10, enabling to categorize results as: normal (< 65) and borderline/clinical (≥ 65). It was assembled cases borderline and clinical to analyze.

The TRF is an instrument similar to the CBCL, but directed to teachers, educationalists and other professionals involved in the children’s development at school. It evaluates the child’s behavior over the prior two months. There are more questions relating to attention problems (26 items) than in the instrument intended for parents and guardians, particularly because teachers perceive attention deficit and hyperactivity problems better. The cutoff procedure is similar to that used with the CBCL.

The survey data that gave rise to this paper yielded a significant Pearson correlation (0.35; p < 0.001) between the CBCL and TRF evaluations of attention disorder.

In the cases examined in this paper, the multidimensional questionnaire of the study was answered exclusively by the children’s mothers. Of the questionnaire answered by the teachers, only the TRF was used. CBCL e TRF’s items related to others behavior problems were answered by the mothers and teachers, but here only the problem attention’s items was analyzed.

The variables used to gauge the ***profiles of child and mother*** were sex, age, mothers’ schooling (≤ 7 years of education versus that used to correspond to an incomplete primary grade of education in Brazil and > 7 years of education), whether or not the mother was a restless child/teenager (used as a proxy for behavior learned socially or inherited genetically by the child), and the child’s intelligence quotient (IQ). The latter was measured by the Wechsler Intelligence Scale for Children (WISC-III), which comprises 13 sub-tests that make up total IQ. When subdivided, it makes it possible to evaluate verbal IQ (relating to verbal comprehension) and execution IQ (perceptual organization and visual processing, planning capacity, non-verbal learning, and skills for thinking and manipulating visual stimuli quickly). The test was applied complete to 26 children from the sample, and to the rest of the sample in reduced format (comprising two sub-tests – vocabulary and cubes). A Pearson correlation coefficient of 0.85 was obtained between the reduced test and total IQ, and 0.88 and 0.83 for verbal IQ and execution IQ, respectively.

The variables that evaluate the child’s and mother’s ***present family environment*** were: a) social support – whether there are people with whom the mother feels comfortable and can talk about almost anything; b) whether or not the mother had got drunk in the prior year; c) the family’s overall functioning, as evaluated by the General Functioning Scale of the McMaster Family Assessment Device [27], which includes 12 questions: it is difficult to plan family activities because of discord; in times of crisis, you can go to each other for help; you cannot talk in the family about the sadness they feel; each person is accepted for what they are; talking about fears or worries is avoided; people show their feelings for each other; bad feelings in the family; feeling accepted for what you are; difficulty of taking decisions in the family; being able to take decisions; not getting on well together; and trusting one another. Response options ranged from “totally agree” to “totally disagree” (1–5 points), with higher scores meaning better overall family functioning. For the purposes of this paper, precarious family functioning is given by results in the range of one standard deviation below the mean, and regular or good functioning above that level. In the study that gave origin to this article, the scale was adapted transculturally to Portuguese, following the steps proposed by [28] (data not yet published). The Cronbach alpha observed was 0.81; d) life events in the prior year, as reflected in 13 items relating to stressors that arose in the child’s family context in the prior year: parents or guardians were or became unemployed; there were or are serious financial problems in the family; the family is living or has lived in overcrowded conditions, with no room; a relative was charged or arrested; the child’s father, mother or sibling died; a close relative of the child’s died; problems with alcohol or drugs occurred in the family; arguments involving the children occurred in the family; the child’s parents separated or divorced; the child’s father and/or mother remarried; the child became very ill and needed medical care; the child was hospitalized; and the child received very bad, unexpected news, such as the death or serious illnesses of someone they loved. In order to build the score, the items were totaled and divided by the total number of valid items answered, multiplied by 13; and e) an adversity indicator, based on the study by Rutter [9]. The items that go to make up this indicator are shown in Table 1. The score is calculated as the ratio between the sum of the items (present = 1/absent = 0) and the valid items answered, multiplied by 5.

**Table 1:** Family adversity indicator

*Family socioeconomic status*	Uses the economic classification criteria of the *Associação Brasileira de Empresas de Pesquisa* (ABEP). It estimates consumer capacity on the basis of indicators such as color television set, bathroom, automobile, monthly-paid maid, duplex refrigerator or freezer, and head of family’s schooling. Strata A, B and C correspond to the upper- and middle-income classes, and D and E to the lower-income classes.
*Adopted child*	Yes or not.
*Number of siblings living in the same household*	Yes (3 or more) or not.
*Child witnessed physical and verbal violence between the parents/guardians*	Gauged by the Conflict Tactics Scales (CTS-PC) developed by Straus [29] and adapted to Portuguese by Hasselmann & Reichenheim [30]. It analyzes the following tactics used at moments of interparental conflict in the prior year: a) verbal aggression between the parents, assessed by acts such as swearing or insulting, sulking, crying, doing things to spite, smashed, hit or kicked things; b) physical violence, as in throwing something at the other person, pushing, slapping or spanking; and c) severe physical violence, identified as punching, kicking, hitting or trying to hit with objects, beating, and threatening to use or actually using a knife or gun. One affirmative item in each of the sub-scales means witnessing interparental violence.
*Minor maternal psychiatric disorder*	Evaluated by the Self-Reported Questionnaire (SRQ20) [31], which measures for the existence of psychic suffering, such as symptoms of depression, anxiety and psychosomatic conditions including headaches, insomnia and so on. Validated in Brazil by Mari & Williams [32], the scale consists of 20 dichotomous questions, with 7 or more items present indicating psychic suffering.

The last block of variables investigated reflects ***the mothers’ condition during pregnancy and childbirth***. The following concerns were addressed: whether the pregnancy was a peaceful time for the mother or was marked by discord and arguments; whether or not she used alcohol, tobacco and other drugs, such as tranquilizers and illegal drugs while pregnant; and whether the child had any congenital or neurological problem or any kind of anomaly at birth.

### Data analysis

The explanatory variables for ADHD were analyzed using logistic regression models. The analysis procedures were followed, using the response variable rated by teacher (TRF) and by mother (CBCL). Variables in the univariate models that were significant at the 10% level entered the selection process for the multiple model. Inclusion of variables in selection for the multiple model was hierarchical [33]: distal level – information relating to the profile of child and mother; intermediate level – present family environment; and proximal level – maternal conditions during pregnancy and childbirth.

Exemplifying the use of the hierarchical model, Leech et al. [34] investigated the effect of children’s exposure to some substances (for example, alcohol) on the occurrence of attention problems and impulsivity. These authors punctuate the following block of variables: maternal characteristics, environmental characteristics, current and prenatal use of substances. Leech et al. [34] consider that the hierarchical levels were based on presupposition that the information relating to the profile of child and mother exert less direct influence on child's development (distal level) and also that maternal conditions during pregnancy and childbirth has more direct influence on child’s development (proximal level).

Within each block, the criterion for selecting variables was to withdraw the variable with the least significant effect from the model sequentially until all the effects present in the model were significant to the 5% level.

The information regarding design of the sample was considered in all the analyses (model fit and tests of association). Accordingly, weights were introduced to correct the point measures, and adjustments were also made for the accuracy estimates. For model fit, the library survey function of R version 2.11.1 software was used.

This project was approved by the research ethics committee of Brazil’s national school of public health (ENSP/FIOCRUZ). The school directors and parents signed a declaration of free and informed consent.

## Results

The 370 children studied can be profiled as follows: 6 to 13 years old (mean 7.9 and SD = 1.1); 50.8% boys; 32.4% identified by guardians as white; 67.3% black/brown and 0.3% yellow/indigenous; 56.8% living with both parents, 25% with only one of them, 17.4% with father and stepmother or mother and stepfather, and 0.8% living with other relatives. Mean members per household was 4.7 (SD = 1.4); 67.7% of mothers reported not completing fundamental education; 5.2% of families had monthly per capita income of less than one minimum wage (R$ 300.00 in 2005), and 70.8%, up to half a minimum wage. IQ ranged from 70 to 140 (mean = 91.8; SD = 13.2).

Prevalence of ADHD among the children studied was 13.3% (6.2% borderline and 7.1% clinical) when the informant was the mother, and 9.2% (3.8% borderline and 5.4% clinical) when teachers reported the disorder. The Kappa between the parents’ and teachers’ evaluations was 0.12, while prevalence-adjusted Kappa was 0.60.

Table 2 shows the univariate analyses of the association between variables of the different hierarchical blocks and mother-reported ADHD. Of the profile-related variables, mother’s schooling and child’s IQ showed significant associations with ADHD. Thus, the likelihood of ADHD in a child whose mother failed to complete fundamental schooling is 2.6 times more than in a child whose mother completed fundamental or higher education. The likelihood of a child’s having ADHD decreases as intelligence quotient rises. Older children seem to be more likely to present the disorder, although this result did not attain the statistical significance proposed in the study.

**Table 2:** Prevalence, odds ratios, univariate analysis of the logistic regression models with mother-reported outcome (CBCL)

**Profile of child and mother**	**Levels**	**Prev. (CI95%)**	**OR (CI95%)**
Sex	Male (N = 188)	15.4 (10.7 - 21.8)	1.48 (0.79 - 2.74)
	Female (N = 182)	11.0 (6.8 - 17.3)	1.00
Mother’s schooling	≤7 years of education (N = 250)	16.0 (11.2 - 22.4)	**2.64**^**c **^**(1.43 - 4.88)**
	>7 years of education (N = 119)	6.7 (3.9 - 11.4)	1.00
Mother restless/hyperactive in childhood	Yes (N = 154)	16.9 (11.3 - 24.6)	1.60 (0.87 - 2.95)
	No (N = 177)	11.3 (7.2 - 17.3)	1.00
Age	-	-	1.35^a^ (0.98 - 1.86)*
IQ	-	-	**0.98**^**c **^(0.96 - 0.99)*
**Present family environment**			
Family functioning	Precarious (N = 52)	30.8 (19.4 - 45.0)	**3.63**^**d **^**(1.95 - 6.78)**
	Fair / good (N = 285)	10.9 (7.5 - 15.6)	**1.00**
Social support	No (N = 82)	23.1 (13.2 - 37.3)	**2.56**^**b **^**(1.21 - 5.43)**
	Yes (N = 286)	10.5 (7.3 - 14.9)	**1.00**
Mother got drunk	Yes (N = 41)	24.5 (14.4 - 38.5)	**2.39**^**b **^**(1.13 - 5.08)**
	No (N = 327)	11.9 (8.2 - 17.1)	1.00
Life events (prior 12 months)	-	-	**1.30**^**d **^(1.14 - 1.48)*
Adversity indicator	-	-	**1.55**^**c **^(1.22 - 1.97)*
**Mother’s situation during pregnancy and childbirth**			
**Pregnancy**			
Discord	Yes (N = 148)	23.6 (15.9 - 33.6)	**4.54**^**d **^**(2.16 - 9.57)**
	No (N = 220)	6.4 (3.7 - 10.7)	**1.00**
Alcohol use	Yes (N = 56)	21.4 (11.8 - 35.6)	**2.03**^**b **^**(1.13 - 3.65)**
	No (N = 313)	11.8 (8.8 - 15.7)	1.00
Tobacco use	Yes (N = 74)	17.5 (10.3 - 28.2)	1.52 (0.82 - 2.84)
	No (N = 295)	12.2 (8.8 - 16.8)	1.00
Other drug use	Yes (N = 10)	20.0 (5.1 - 54.0)	1.69 (0.37 - 7.75)
	No (N = 357)	12.9 (9.4 - 17.4)	1.00
Child had some health problem at birth	Yes (N = 34)	17.8 (6.0 - 42.6)	0.67 (0.17 - 2.65)
	No (N = 332)	12.6 (8.8 - 17.9)	1.00

^a^p < 0.10; ^b^p < 0.05; ^c^p < 0.01; ^d^p < 0.001 - Wald Test; *Calculated by applying exponential on model’s coefficient estimated; bold OR and CI95% are statistically significant.

Table 2 also shows all the variables relating to the family environment that displayed an association with ADHD. Precariously functioning families were 3.6 times more likely to have children with the disorder; mothers’ who reported not feeling socially supported were 2.6 times more likely to have children with ADHD than those who did have such support; children of mothers who had drunk alcohol to the point of drunkenness in the prior year were 2.4 times more liable to ADHD than those whose mothers had not; with each additional stressor in the child’s family context, the likelihood of ADHD increased by approximately 30%; and each additional adversity in a child’s life produced an increase of nearly 55% in the likelihood of its developing the disorder.

As regards maternal conditions during pregnancy and childbirth, mothers who had fights or who consumed alcohol in that period had odds ratios of 4.5 and 2, respectively.

Table 3 shows the variables after selection of the final hierarchical multiple model arrived at after three stages: a) model 1: fitted with only the distal level variables with p < 0.10; mothers’ schooling was significant (p = 0.003); b) model 2: fitted with the mothers’ schooling and the intermediate level variables. In the second model, the following variables continued significantly associated with mother-reported ADHD: family functioning (p = 0.012), social support (p = 0.039) and life events (p = 0.005); and c) model 3: variables significant in the previous model, plus the proximal level variables. The final result is shown in Table 3.

**Table 3:** Hierarchical multiple model with mother-reported outcome (CBCL)

**Factors**	**Levels**	**OR**	**CI95%**
Family functioning	Precarious	**2.14**	**1.11 - 4.13**
Social support	No	**2.35**	**1.10 - 5.02**
Life events, prior 12 months	-	1.22*	**1.04 - 1.43**
Pregnancy	Discord and fights	**3.77**	**1.82 - 7.84**

*Calculated by applying exponential on model’s coefficient estimated; bold OR and CI95% are statistically significant.

Comparing the crude odds ratios (Table 2) and odds ratios adjusted for the other variables (Table 3), what is conspicuous is the decreased effect of family functioning and fights during pregnancy. The family environment proved important, because it associates with ADHD in children when the disorder is reported by the mother.

Table 4 shows the same three blocks of factors as in Table 2, except that the criterion is teacher-reported ADHD. The variables significantly associated with the outcome occur only in the child profile block: male children were 4.2 times more likely than female children to have the disorder; a one-year increase in age associated with 43% greater likelihood of ADHD developing; and children with higher IQs were less likely to have the symptoms of the disorder. Mothers’ lack of schooling was a factor showing a possible association with the disorder (p < 0.10).

**Table 4:** Prevalence, odds ratios, univariate analysis of the logistic regression models with teacher-reported outcome (TRF)

**Profile of child and mother**	**Levels**	**Prev. (CI95%)**	**OR (CI95%)**
Sex	Male (N = 188)	14.4 (9.0 - 22.3)	**4.22**^**b **^**(1.58 - 11.27)**
	Female (N = 182)	3.8 (1.7 - 8.3)	1.00
Mother’s schooling	Fundamental incomplete (N = 250)	10.8 (6.9 - 16.5)	2.28^a^ (0.88 - 5.89)
	Fundamental complete and higher (N = 119)	5.1 (2.2 - 11.2)	1.00
Mother restless / hyperactive in childhood	Yes (N = 154)	9.8 (5.2 - 17.6)	1.64 (0.66 - 4.06)
	No (N = 177)	6.2 (3.1 - 12.0)	
Age	-	-	**1.43**^**b **^(1.13 - 1.82)*
IQ	-	-	**0.95**^**b **^(0.92 - 0.98)*
**Present family environment**			
Family functioning	Precarious (N = 52)	11.5 (5.7 - 21.9)	1.26 (0.48 - 3.24)
	Fair / good (N = 285)	9.5 (5.9 - 14.9)	1.00
Social support	No (N = 82)	9.8 (4.4 - 20.1)	1.08 (0.47 - 2.49)
	Yes (N = 286)	9.1 (6.2 - 13.2)	1.00
Mother got drunk	Yes (N = 41)	7.4 (2.4 - 20.5)	0.76 (0.22 - 2.62)
	No (N = 327)	9.5 (6.3 - 14.0)	1.00
Life events, prior 12 months	-	-	0.90 (0.74 - 1.08)*
Adversity indicator	-	-	1.00 (0.71 - 1.41)*
**Mother’s situation during pregnancy and childbirth**			
**Pregnancy**			
Discord	Yes (N = 148)	8.8 (5.0 - 15.1)	0.92 (0.40 - 2.09)
	No (N = 220)	9.5 (5.6 - 15.7)	1.00
Alcohol use	Yes (N = 56)	8.9 (4.0 - 18.7)	0.96 (0.37 - 2.50)
	No (N = 313)	9.3 (6.0 - 14.0)	1.00
Tobacco use	Yes (N = 74)	9.5 (4.4 - 19.3)	1.04 (0.37 - 2.95)
	No (N = 295)	9.2 (5.7 - 14.5)	1.00
Other drug use	Yes (N = 10)	-	-
	No (N = 357)	9.5 (6.4 - 13.9)	
Child had some health problem at birth	Yes (N = 34)	11.8 (4.7 - 26.7)	0.74 (0.23 - 2.36)
	No (N = 332)	9.0 (5.8 - 13.8)	1.00

^a^p < 0.10; ^b^p < 0.05 - Wald Test; *Calculated by applying exponential on model’s coefficient estimated; bold OR and CI95% are statistically significant.

The prevalences of ADHD measured by the TRF differed markedly by sex (14.4% of boys and 3.8% of girls). The prevalence among girls was quite different from the corresponding mother-reported prevalence (11%); there was closer agreement as regards the boys (15.4%) (Table 4).

From the data in Table 4, a model was fitted with the variables with p < 0.10: sex, age, IQ and mother’s schooling. The final model is shown in Table 5, where only sex and IQ remain as explanatory variables for teacher-reported ADHD. Boys were 4.3 times more likely to display the problem than girls, and a one-point increase in IQ score associated with an approximately 5% lesser likelihood of having the disorder.

**Table 5:** Hierarchical multiple model with TRF-assessed outcome

**Factors**	**Levels**	**OR**	**CI95%**
Sex	Male	**4.31**	**1.62 - 11.50**
IQ	-	0.95*	**0.92 - 0.98**

*Calculated by applying exponential on model’s coefficient estimated; bold OR and CI95% are statistically significant.

In the two models (CBCL and TRF) the interaction terms were tested, but were not significant.

## Discussion

The results show a difference between the percentage of ADHD (borderline/clinical) as reported by parents and by teachers. Mothers reported more in their children than the teachers did (13.3% against 9.2%), and that trend persists even when comparing only the prevalence of clinical cases in the two samples (7.0% against 5.4%). Recent review studies have shown major variations in prevalences (from 0.2% to 26.8%), which relate to the different methodological strategies used in the studies (e.g. evaluation criteria and informants considered), which can affect these measurements of prevalence [35]. A study in Florianópolis, of 1898 schoolchildren from 6 to 12 years old, found 5% of cases in the sample [14]. Rodhe [36] investigated 1022 schoolchildren in Porto Alegre (Rio Grande do Sul), finding a prevalence of 5.8%.

In addition, the ADHD’s percent is expected to vary by information source (parents or teachers). The traditional Kappa found weak agreement between mothers and teachers, although part of that result can be explained by the low frequency of ADHD in the population studied, given that the adjusted Kappa was considered substantial on the criteria of Landis and Koch [37]. Similar results have been found by Wolraich et al. [19] using the traditional Kappa.

Reported percentage de ADHD among boys was similar (mothers, 15.4% and teachers, 14.4%), but quite the opposite was true among girls (11% and 3.8%, respectively), corroborating the findings indicated in the literature that teachers have more difficulty evaluating lack of attention, hyperactivity and impulsiveness among girls, who culturally are expected to be quieter and more introverted [21]. Parents are more likely to recognize symptoms of ADHD in girls, because they compare their daughters with other girls (often with no ADHD symptoms) in their circle of relationships. Teachers, on the other hand, compare girls with ADHD to boys with the same disorder, thus underestimating the former’s symptoms, as the problems are more evident in the boys [1].

The association between precarious family functioning and mother-reported ADHD, as observed in this study, is also reported by other authors. Scahill et al. [38] found more severe children’s symptoms associated with higher levels of family dysfunction. Lange et al. [6] also detected that, in addition to more fragile family functioning, those responsible for children with ADHD also perceived less social support. Edwards et al. [39], however, indicate that it is uncertain whether family dysfunction is a cause or effect of the disorder; after all, a child with ADHD makes for a more difficult climate in the family, and vice-versa. Note that precarious family functioning only displayed an association with ADHD when the informant was the mother, which may indicate that that the ADHD symptoms she reports relate to difficulties she is experiencing in her family.

Child ADHD as seen by the mother is also explained by lack of social support, stressful life events, and a pregnancy marked by conflict. Lange et al. and Counts et al. [5,6] also found that stressful life events were associated with ADHD, and that the accumulation of them is relevant to the emergence of ADHD reported by parents/guardians. Rodriguez and Bohlin [40] found that stress during pregnancy has a relationship with ADHD (as reported by teachers and guardians).

In the teachers’ view, besides male sex, only the child’s IQ showed an association with ADHD, corroborating the finding that teachers tend to evaluate more by reference to neurocognitive considerations [20].

Some variables that showed an association only in the univariate analysis deserve brief mention. One is alcohol use during pregnancy and maternal drunkenness in the months prior to the study. Mick et al. [11] found that exposure to alcohol abuse during pregnancy increased the risk of ADHD in the children, independently of the presence of relatives with ADHD or antisocial disorders. They argue that the presence of ADHD in children may be an additional adverse effect of exposure to alcohol during pregnancy. Adversities also deserve mention as an indicator: many of the factors present in the adversity indicator suggested by Rutter are close in interpretation to the factors contained in the life events indicator, which best represented the emergence of mother-reported ADHD.

Limitations of the study that deserve special mention are the use of retrospective measures (for example, information on the pregnancy and the mothers’ childhood behavior) and a screening instrument directed to behavioral problems in general (and not a diagnostic instrument exclusive to ADHD). The cross-sectional study design precludes evaluating causal relations between the effects analyzed and the outcome. The lack of evaluation by psychiatrists and of information provided by the children themselves (replaced in the study by mothers and teachers) contribute to continuing imprecision as to the presence of this very complex phenomenon in Brazil. The lack of information on co-morbidities parallel to ADHD is another limitation, because the high rate of co-morbidities in childhood is one of the factors that most hinders diagnosis. Biederman [41] in a review article, showed the main comorbidities in children with ADHD were oppositional defiant disorder (60%), conduct disorder (15%), mood disorders (25%) anxiety disorders (28%) and learning disorders (27%). They consider the dificuldade in the ADHD diagnosis due to plenty of comorbidities, their types and frequency. These aspects emphasizes the relevance of future research to include the issue of comorbidity and ADHD.

Lastly, the sample studied in this paper comprised public school pupils only, making it difficult to study effects related to socioeconomic conditions.

In spite the substantial number of children excluded from the analysis, these losses did not constitute a profile differentiated from the study sample, i.e., by sex, age and social class.

## Conclusions

In order to understand the phenomenon better, it is important that more studies should be conducted on the views of different informants in assessing ADHD. Correct diagnosis of the disorder depends on the existence of a clearly defined history of behavioral symptoms and of the harm resulting from those symptoms [18].

## Competing interests

The authors declare that they have no competing interests.

## Authors’ contributions

TOP conducted the literature search and data analysis, and drafted the article. CMS and SGA made a substantial contribution to the methodology and interpretation of results and helped draft the manuscript. All of the authors read and approved the final manuscript.

## Pre-publication history

The pre-publication history for this paper can be accessed here:

http://www.biomedcentral.com/1471-244X/13/215/prepub
